# Mr. Forster's Case of Tumors in the Thorax

**Published:** 1812-11

**Authors:** Thomas Forster

**Affiliations:** Clapton


*To the Editors of the Medical and Physical Journal.*

GENTLEMEN,

THINKING the following account of three large Tumors, which I recently took from out the abdominal cavity of a Chicken, might prove interesting to some of your readers, I submit it for insertion in your Journal.

The bird was a female, and of that variety usually denominated the dunghill fowl. It had been observed for a long time to go lame, in consequence of which its owner took it from among the other poultry, and kept it in the house. Its new situation did not seem to agree with it; it continued to get worse from the period of its domiciliation; the lameness increased, the muscles wasted, and it at length dropped down dead on the hearth.

Being anxious to collect all the specimens of morbid alterations of structure found in animals which have died of disease, I have lately been in the habit of opening the bodies of all which came in my way; and the farmer, knowing this, sent me the defunct as a subject of dissection.

On cutting into the cavity of the thorax, a large quantity of fetid matter, of a yellowish creamy consistence, gushed out from the right side of it; perhaps enough to have filled the bowl of a tobacco-pipe. But the most extraordinary appearances were in the abdomen. All the left side of that cavity was occupied by three large tumors, which had shoved the bowels out of their natural situation. The largest of these was of a roundish form, and lay in front, occasioning great distension. It was about two inches and three quarters in diameter. On making a section of it, I found it to be composed of successive layers of condensed substance, in the interstices of which was deposited a sort of cheese-like matter, of a light brown color; numerous blood-vessels ramified over its surface. Under this was a smaller tumor, of the same shape. The space between these two and the loins, was occupied by a large tumor of a very peculiar figure. It was near eight inches long, and one in diameter, but was folded up somewhat in the form of the Greek letter Ω, if we suppose its two extremities curved upwards: its substance was harder and more compact than that of the other two. The intestines all lay on the right side of the abdomen. By inflation with a blow pipe, the continuity of their canal did not appear to be interrupted, but was quite empty. The stomach was harder, and its coat thicker, than usual: it was filled with small gravel stones, such as birds of this class usually swallow. The liver was large, but in other respects healthy.

In

In most of the domesticated animals that I have dissected, which had died of disease, I have found morbid alterations of structure; but I have never met with any in wild ones, which I have accidentally found dead, but without any marks of having been killed by accident. In a tame daw (*Corvus monedula*), the aorta near the heart was ossified; and the whole heart was ossified in a linnet (*Fringilla Linaria*) which I lately dissected. I could enumerate many more such instances.

*Clapton,* THOMAS FORSTER.

*Feb.* 14, 1812.

